# A novel nutrition-related nomogram for the survival prediction of colorectal cancer-results from a multicenter study

**DOI:** 10.1186/s12986-022-00719-8

**Published:** 2023-01-04

**Authors:** Guo-Tian Ruan, Meng-Meng Song, Kang-Ping Zhang, Hai-Lun Xie, Qi Zhang, Xi Zhang, Meng Tang, Xiao-Wei Zhang, Yi-Zhong Ge, Ming Yang, Li-Chen Zhu, Han-Ping Shi

**Affiliations:** 1 Department of Gastrointestinal Surgery/Department of Clinical Nutrition, Beijing Shijitan Hospital, Capital Medical University, 10 Tie Yi Road, Beijing, 100038 China; 2 Key Laboratory of Cancer FSMP for State Market Regulation, Beijing, 100038 China; 3 Department of Immunology, School of Preclinical Medicine, Guangxi Medical University, Nanning, China

**Keywords:** Colorectal cancer, Nomogram, TNM stage, Nutrition, Overall survival

## Abstract

**Background:**

Precisely predicting the short- and long-term survival of patients with cancer is important. The tumor-node-metastasis (TNM) stage can accurately predict the long-term, but not short-term, survival of cancer. Nutritional status can affect the individual status and short-term outcomes of patients with cancer. Our hypothesis was that incorporating TNM stage and nutrition-related factors into one nomogram improves the survival prediction for patients with colorectal cancer (CRC).

**Method:**

This multicenter prospective primary cohort included 1373 patients with CRC, and the internal validation cohort enrolled 409 patients with CRC. Least absolute shrinkage and selection operator regression analyses were used to select prognostic indicators and develop a nomogram. The concordance (C)-index, receiver operating characteristic (ROC) curve, and decision curve analysis (DCA) were used to assess the prognostic discriminative ability of the nomogram, TNM stage, Patient-Generated Subjective Global Assessment (PGSGA), and TNM stage + PGSGA models. The overall survival (OS) curve of risk group stratification was calculated based on the nomogram risk score.

**Results:**

TNM stage, radical resection, reduced food intake, activities and function declined, and albumin were selected to develop the nomogram. The C-index and calibration plots of the nomogram showed good discrimination and consistency for CRC. Additionally, the ROC curves and DCA of the nomogram showed better survival prediction abilities in CRC than the other models. The stratification curves of the different risk groups of the different TNM categories were significantly different.

**Conclusion:**

The novel nomogram showed good short- and long-term outcomes of OS in patients with CRC. This model provides a personalized and convenient prognostic prediction tool for clinical applications.

**Supplementary Information:**

The online version contains supplementary material available at 10.1186/s12986-022-00719-8.

## Background

Colorectal cancer (CRC), with high morbidity and mortality, is one of the most prevalent malignant neoplasms worldwide. In 2018, global cancer data showed that CRC ranked third in morbidity and second in mortality [1]. At present, the tumor-node-metastasis (TNM) staging system is still the most practical and widely used cancer classification and prognostic prediction system for CRC [2]. However, this system cannot provide all prognostic information, and patients with the same histopathological stage may have significant differences in clinical outcomes [3]. Therefore, it is necessary to identify clinicopathological features that can affect the prognosis of patients with CRC rather than TNM staging.

Malnutrition is more prevalent (approximately 30–60%) [4] in patients with CRC than in patients with non-gastrointestinal cancer [5]. This is due to the combined effects of cancer progression, host response to tumors, anticancer therapies, and direct effects of intestinal obstruction and malabsorption [6, 7]. Malnutrition can also adversely affect the clinical outcomes of patients with cancer, such as overall survival (OS) and tolerance to chemotherapy [8, 9]. The Patient-Generated Subjective Assessment (PGSGA), adapted from the Subjective Global Assessment, is widely used in clinical and academic research as a reference for evaluating the nutritional status of patients with cancer [10]. The PGSGA had higher clinical benefits in assessing the nutritional status of patients with malignant neoplasms in their digestive system [11]. More specifically, Read et al. found PGSGA to be useful as a prognostic model to predict OS in patients with cancer [12]. Yang et al. also found that malnutrition was associated with a lower OS in patients with CRC using PGSGA [11].

The nomogram is an essential part of the decision-making process in modern medical practice. By combining and exploring important factors for tumor prognosis, nomograms have been recognized as a reliable tool for quantifying risk [13, 14]. The nomogram produces numerical probabilities of clinical events, such as OS, by creating intuitive graphs of statistical predictive models [15]. Currently, the nomogram can produce more accurate predictions for cancers than the traditional TNM staging system [16–18]. Yamamoto et al. reported that this might be caused by different prognostic factors, including nutritional status [19]. Furthermore, nutritional status affects the short-term survival of patients. TNM staging is crucial for the accurate prediction of long-term outcomes, but it cannot accurately predict short-term outcomes [13]. Their combination may enhance the accuracy of short-term survival predictions. Therefore, for the first time, we incorporated nutrition-related prognostic indicators into clinicopathological prognostic factors (including the TNM staging system) to establish and validate a novel prognostic nomogram model and compared different established models to screen for the most applicable one. Finally, we investigated the potential clinical value of the nomogram in CRC.

## Methods

### Patient screening

A prospective cross-sectional study was conducted from multiple Chinese medical centers (including Foshan First People's Hospital, Fujian Provincial Cancer Hospital, Affiliated Cancer Hospital of Zunyi Medical College, The fourth Hospital of Harbin Medical University, Bethune First Hospital of Jilin University, The First Affiliated Hospital of Sun Yat-sen University, Cancer Hospital, Chinese Academy of Medical Sciences, and Chongqing Daping Hospital) that collected data on patients with CRC from July 2013 to December 2018. The inclusion criteria were as follows: (1) pathological diagnosis of primary CRC, (2) age ≥ 18 years, (3) more than 48 h of hospitalization, and (4) signed informed consent. There were no strict exclusion criteria. After excluding 123 cases with missing data (including 42 cases with missing total protein and 81 cases with missing albumin information data), a total of 1373 patients with CRC were included in this study. In addition, we randomly selected 30% of these patients as an internal validation dataset. Additional file 1 presents the detailed information for selection in a flowchart. This study was in accordance with the Declaration of Helsinki and was approved by the institutional review board of each hospital (registration number: ChiCTR1800020329).

### Data collection

This study collected patients’ demographic and clinicopathological characteristics, including age, sex, lifestyle (smoking status, alcohol consumption, and tea consumption), comorbidity (liver cirrhosis, hypertension, diabetes), family history of cancer, TNM stage, radical resection, nutrition-related information (weight loss, reduced food intake, activities and function declined, body mass index [BMI], and nutritional intervention), and serum total protein and serum albumin levels. Weight loss (unintentional) was estimated based on the comparison between the patient’s weight at the time of admission (wearing a light hospital gown and without shoes) and the usual weight (the patient’s usual measurement and self-description). Weight loss of not less than 2% in the last 6 months or 2 weeks was defined as the presence of weight loss (yes or no). Reduced intake was associated with reduced food intake and eating problems. It was assessed by comparing the change in food intake between the current and past months. Eating problems were evaluated by the patient’s self-description of the physical symptoms, including nausea, vomiting, diarrhea, and constipation. The decline in activities and functions was estimated by the change within the past month. Patient self-reported presence of reduced dietary intake and decreased physical activity was defined as reduced food intake (yes or no) and activities and function declined (yes or no), respectively. The BMI was calculated as follows: BMI = body mass (kg)/height (m)^2^.

### Nutrition assessment and survival outcome

The PGSGA criteria mainly included two sections: the patient’s self-assessment section (Box 1, Weight change in 1 or 6 months; Box 2, Food intake changing during the past month; Box 3, Symptoms that affected food intake during the past 2 weeks; and Box 4, Activities and function changing over the past month) and professional assessment (diseases, metabolic demand, and physical examination). The scored PGSGA was classified into three categories based on the scores: A (0–3), well nourished; B (4–9), moderately/suspected malnourished; and C (> 9), severely malnourished.

The patient follow-up data were carried out through outpatient follow-up, hospitalization records, and regular telephone follow-up. The primary survival outcome in this study we observed was the overall survival (OS) of patients with CRC. OS was defined as the time from the initial cancer diagnosis to death or the last censored.

### Statistical analyses

**Fig. 1:**
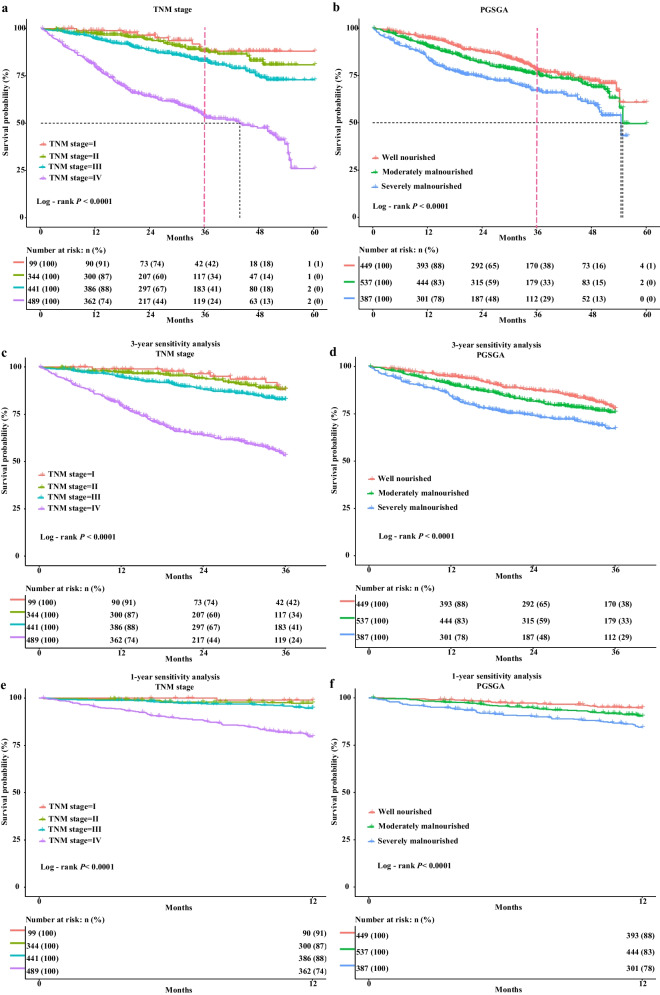
Identification of prognostic indicators in CRC using LASSO and Cox regression analysis. **A** LASSO coefficient profiles of 16 indicators in CRC; **B** Plots of the cross-validation error rates. Each dot represents a lambda value along with error bars to give a confidence interval for the cross-validated error rate; **C** Multivariable Cox regression identified five prognostic indicators in the primary cohort. Notes: CRC, colorectal cancer; LASSO: least absolute shrinkage and selection operator; TNM stage, tumor-node-metastasis stage

Continuous quantitative variables are represented as mean ± standard deviation, whereas categorical variables are represented by the number of patients (percentage). We used Student’s t-test to analyze continuous variables conforming to a normal distribution and nonparametric tests (Mann–Whitney or Kruskal–Wallis) for variables not conforming to a normal distribution. Overall survival (OS) was calculated using the Kaplan–Meier method. In addition, we performed a sensitivity analysis by KM survival curves (univariable survival analysis) excluding patients who died within 1 year to further confirm our prediction of survival. Our model was constructed based on TNM stage system and PGSGA diagnostic tools using the least absolute shrinkage and selection operator (LASSO) regression method for dimensionality reduction to select the optimal prognostic parameters—TNM stage, radical resection, reduced food intake, activities and function declined, and low albumin level from all relevant parameters (Fig. 1A, B). The concordance (C)-index and calibration plots were used to judge the survival prediction ability and accuracy of the constructed model. Prognostic receiver operator characteristic (ROC) curves and decision curve analysis (DCA) curves were used to judge the prognostic predictive ability of different models, including Nomogram, TNM stage, PGSGA, and TNM stage + PGSGA. We divided the nomogram score into a high-risk group and a low-risk group according to the cut-off value of the nomogram score (Additional file 2, score: ≥ 86.08 vs. < 86.08).

Univariable and multivariable Cox regression analyses were used to analyze the OS of the nomogram model for CRC. The adjusted model for multivariable survival analysis included TNM stage, radical resection, reduced food intake, activities and function declined, and serum albumin. Hazard ratios (HRs) and 95% confidence intervals (CIs) were used to evaluate the contribution and reduce clinical bias. The two tails of *P* < 0.05 indicated statistical significance. All the analyses above were performed and detected using the Statistical Package for the Social Sciences (SPSS) version 20.0 (SPSS Inc., Chicago, IL) and R version 3.6.2.

## Results

### Clinicopathological characteristics

We collected patients’ demographic and clinicopathological information from the two cohorts. The primary cohort comprised 1,373 patients, of whom 58.5% were men (n = 803). The median follow-up time for patients with CRC in the primary cohort was 34.1 months. A total of 329 deaths occurred during the follow-up period. Among the tumor stages, 99 patients (7.2%) were in stage I, 344 (25.1%) in stage II, 441 (32.1%) in stage III, and 489 (35.6%) in stage IV. Based on the PGSGA criteria, 924 (67.3%) patients were diagnosed with malnutrition, and 449 (32.7%) patients were well nourished (Table 1).

**Table 1:** Demographic and clinicopathological characteristics

Demographic or clinicopathological characteristic	Primary cohort (N = 1373)	Internal validation cohort (N = 409)	*P* value
	No. of patients (%)	No. of patients (%)	
*General information*			
Age, > 65 years	421 (30.7)	122 (29.8)	0.795
Sex, male	803 (58.5)	247 (60.4)	0.528
Smoking status, yes	517 (37.7)	159 (38.9)	0.698
Alcohol consumption, yes	251 (18.3)	72 (17.6)	0.811
Tea consumption, yes	374 (27.2)	127 (31.1)	0.149
Comorbid disease(s)			0.932
0	877 (63.9)	261 (63.8)	
1	377 (27.5)	109 (26.7)	
2	95 (6.9)	32 (7.8)	
3 or more	24 (1.7)	7 (1.7)	
Family history of cancer, yes	193 (14.1)	58 (14.2)	1
TNM stage			0.378
I	99 (7.2)	33 (8.1)	
II	344 (25.1)	116 (28.4)	
III	441 (32.1)	116 (28.4)	
IV	489 (35.6)	144 (35.2)	
Radical resection, yes	539 (39.3)	163 (39.9)	0.874
*Nutrition related information*			
PGSGA Criteria (nutritional index)			0.365
Well nourished (0–3)	449 (32.7)	149 (36.4)	
Moderately malnourished (4–8)	537 (39.1)	149 (36.4)	
Severely malnourished (> 9)	387 (28.2)	111 (27.1)	
Weight loss, yes	685 (49.9)	207 (50.6)	0.842
Reduced intake, yes	588 (42.8)	163 (39.9)	0.874
Activities and function declined, yes	473 (34.5)	143 (35.0)	0.895
BMI, kg/m^2^			0.676
< 18.5	153 (11.1)	40 (9.8)	
18.5–23.9	770 (56.1)	235 (57.5)	
24–27.9	367 (26.7)	114 (27.9)	
≥ 28	83 (6.0)	20 (4.9)	
Nutritional intervention, yes	334 (24.3)	99 (24.2)	1
Serum total protein, < 60 g/L	263 (19.2)	71 (17.4)	0.456
Serum albumin, < 35 g/L	373 (27.2)	92 (22.5)	0.608

TNM stage, tumor-node-metastasis stage; PGSGA, patient generated subjective global assessment; BMI, body mass index

The internal validation cohort was a total of 409 patients with 60.4% of them being male (n = 247). The median follow-up time for patients with CRC in the internal validation cohort was 34.9 months. A total of 98 deaths occurred during the follow-up period. Among the tumor stages, 33 patients (8.1%) were in stage I, 116 (28.4%) in stage II, 116 (28.4%) in stage III, and 144 (35.2) in stage IV. Based on the PGSGA criteria, 260 (63.6%) patients were diagnosed with malnutrition, and 149 (36.4%) patients were well nourished (Table 1).

### Survival curves and sensitivity analyses of tumor-node-metastasis stage and Patient-Generated Subjective Global Assessment

**Fig. 2:**
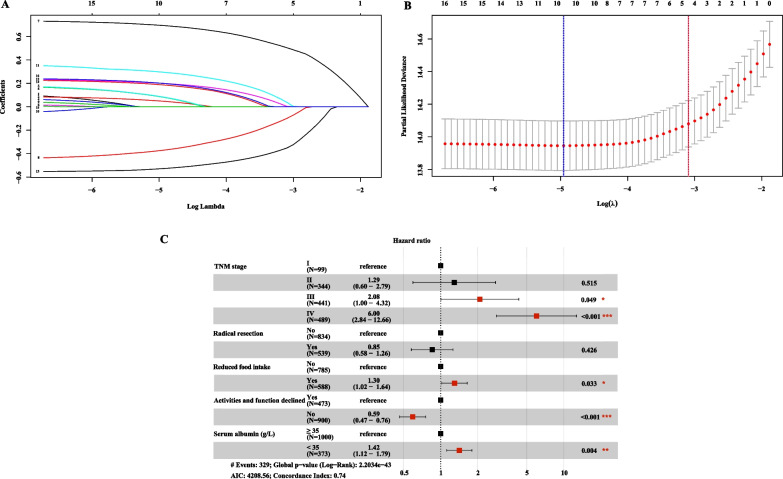
The Kaplan–Meier survival curves and sensitivity analyses of CRC OS. **A** Survival curves of TNM stage; **B** Survival curves of PGSGA; **C** 3-year sensitivity analysis of TNM stage; **D** 3-year sensitivity analysis of PGSGA; **E** 1-year sensitivity analysis of TNM stage; **F** 1-year sensitivity analysis of PGSGA. Notes: CRC, colorectal cancer; OS, overall survival; TNM stage, tumor-node-metastasis stage; PGSGA, patient generated subjective global assessment

Our survival curve showed that TNM stage and PGSGA had a statistically significant difference in the prognosis of CRC (all *P* < 0.001). However, we also found that the prognostic prediction ability between TNM stage I and II was poor in CRC 3 years after the patients were diagnosed with cancer. In contrast, the prognostic prediction ability of PGSGA was relatively better than that of the TNM stage (Fig. 2A, B). Similarly, our sensitivity analysis also found consistent results in patients with cancer for 3 years. In addition, the 1-year sensitivity analysis showed that the prediction ability was poor among TNM stages I, II, and III (Fig. 2C–F).

### Development of nomogram

**Fig. 3:**
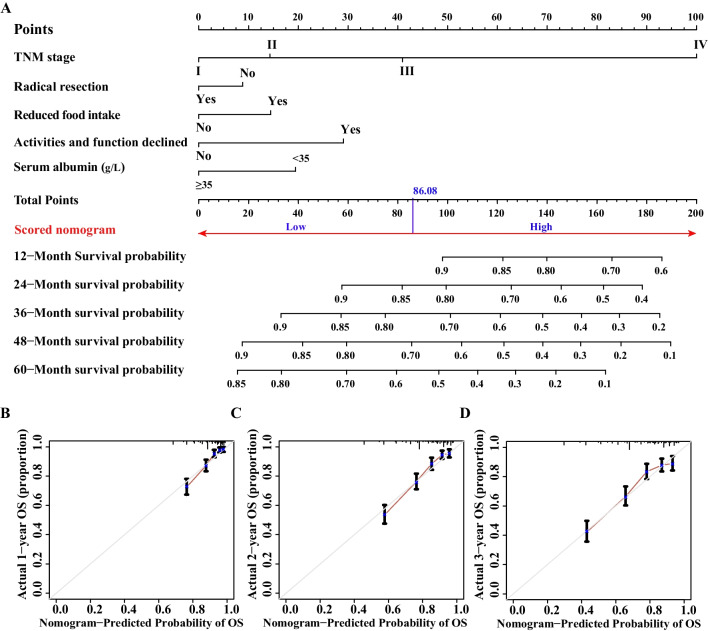
Development of the nomogram model in CRC. **A** Prognostic nomogram predicting 1-, 2-, 3-, 4-, and 5-year OS probability using the five prognostic indicators. **B**–**D** Calibration curve of the nomogram predicting the 1-, 2-, and 3-year probability of OS in CRC. Notes: CRC, colorectal cancer; OS, overall survival

The LASSO showed that the TNM stage, radical resection, reduced food intake, activities and function declined, and low albumin level were selected as optimal indicators. Additionally, multivariable Cox regression analyses also suggested that these indicators were associated with CRC OS and were identified as prognostic indicators for the new nomogram model (Fig. 1C). Finally, we took advantage of these prognostic indicators to build a prognostic nomogram model of CRC (Fig. 3A), and the total points were obtained by adding the scores of each indicator. The greater the number of points obtained, the greater the risk and the lower the probability of survival. This nomogram gives a more accurate prediction of survival. The calibration plots showed that the nomogram had an excellent survival prediction consistency for patients with CRC (Fig. 3B, D).

### Analysis and comparison of the prognostic value among different models

**Fig. 4:**
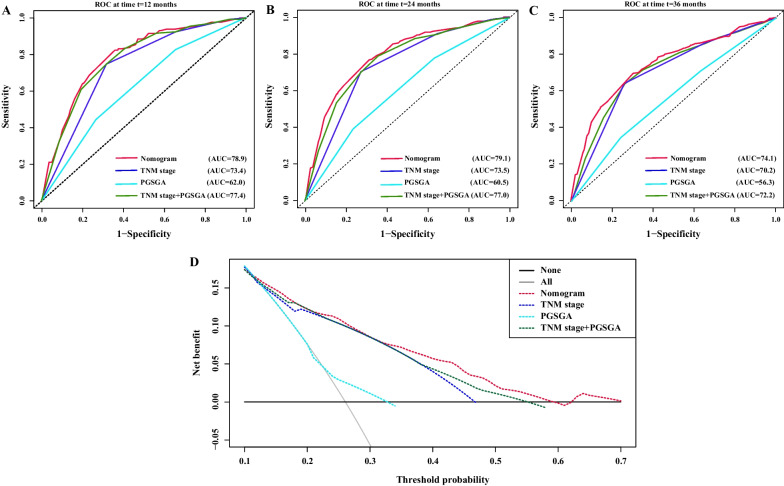
Analysis and comparison of the prognostic value among different models. **A** The time-dependent ROC curves for 12-, 24-, and 36-month CRC OS in the primary cohort; **B** DCA of the nomogram model. Nomogram model (red line), TNM staging system model (blue line), PGSGA model (cyan line), and TNM staging system combined with PGSGA model (green line). Notes: CRC, colorectal cancer; OS, overall survival; ROC, receiver operating characteristic curve; TNM Stage, tumor-node-metastasis stage; PGSGA, patient generated subjective global assessment

We found that TNM staging was a weak predictor of survival in the first three years of patients with CRC, so we compared the predictive value of our constructed model, TNM staging, and PGSGA for survival in the first three years. The C-indexes of the different models were as follows: 0.74 in nomogram model (95% CI, 0.72–0.77), 0.70 in TNM stage model (95% CI, 0.67–0.72, comparable *P* < 0.001), 0.58 in PGSGA model (95% CI, 0.55–0.61, comparable *P* < 0.001), and 0.73 in TNM stage + PGSGA model (95% CI, 0.67–0.76, comparable *P* = 0.004) (Additional file 3: Table S1). The 1-, 2-, and 3-year time-dependent ROC curves showed that the area under the curve (AUC) of the nomogram model (1-year, AUC = 78.9; 2-year, AUC = 79.1; 3-year, AUC = 74.1) was higher than that of TNM stage(1-year, AUC = 73.4; 2-year, AUC = 73.5; 3-year, AUC = 70.2), PGSGA(1-year, AUC = 62.0; 2-year, AUC = 60.5; 3-year, AUC = 56.3), and TNM stage + PGSGA models (1-year, AUC = 77.4; 2-year, AUC = 77.0; 3-year, AUC = 72.2) (Fig. 4A–C). Finally, the nomogram model’s reliability and benefit were evaluated by DCA, which indicated that the nomogram model had a better effect in predicting the 3-year survival of patients with CRC than the other models (Fig. 4D).

### Validation of nomogram based on internal validation cohort

**Fig. 5:**
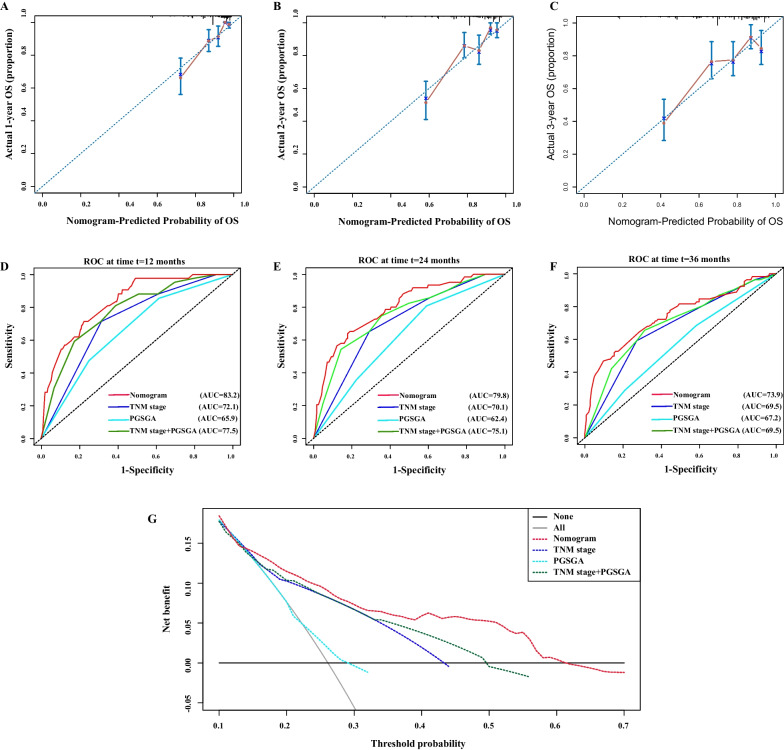
Validation of the prognostic value of nomogram in internal validation cohort. **A**–**C** The 1-, 2-, and 3- year calibration plots of nomogram; **D**–**F** The time-dependent ROC curves for 12-, 24-, and 36-month CRC OS; **G** DCA of the nomogram model. Nomogram model (red line), TNM staging system model (blue line), PGSGA model (cyan line), and TNM staging system combined with PGSGA model (green line). Notes: CRC, colorectal cancer; OS, overall survival; ROC, receiver operating characteristic curve; TNM Stage, tumor-node-metastasis stage; PGSGA, patient generated subjective global assessment

The calibration plots of nomogram showed a good survival prediction consistency of patients with CRC (Fig. 5A–C). We also validated the prognostic value of the nomogram in the internal validation cohort. The C-indexes of the different models were as follows: 0.75 in nomogram model (95% CI, 0.72–0.77), 0.68 in TNM stage model (95% CI, 0.63–0.73, comparable *P* < 0.001), 0.59 in PGSGA model (95% CI, 0.53–0.64, comparable *P* < 0.001), and 0.71 in TNM stage + PGSGA model (95% CI, 0.66–0.77, comparable *P* = 0.026) (Additional file 3: Table S1). The 1-, 2-, and 3-year time-dependent ROC curves showed that the AUC of the nomogram model (1-year, AUC = 83.2; 2-year, AUC = 79.8; 3-year, AUC = 73.9) was higher than that of TNM stage (1-year, AUC = 72.1; 2-year, AUC = 70.1; 3-year, AUC = 69.5), PGSGA (1-year, AUC = 65.9; 2-year, AUC = 62.4; 3-year, AUC = 67.2), and TNM stage + PGSGA models (1-year, AUC = 77.5; 2-year, AUC = 75.1; 3-year, AUC = 69.5) (Fig. 5D–F). Finally, the nomogram model’s reliability and benefit were evaluated by DCA, which indicated that the nomogram model had a better effect in predicting the 3-year survival of patients with CRC than the other models (Fig. 5G).

### Risk group stratification of nomogram in primary cohort

**Fig. 6:**
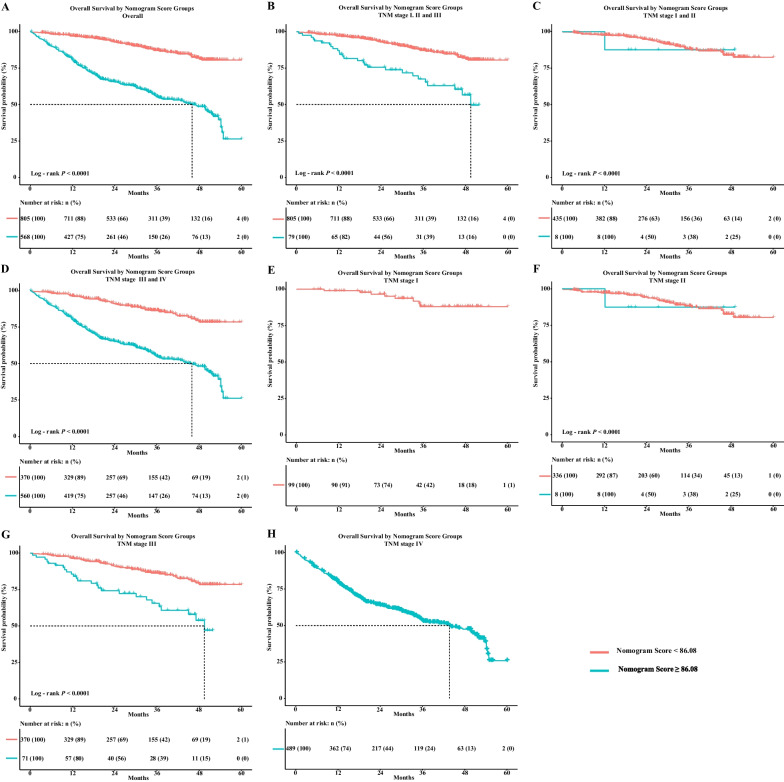
Risk group stratification within each TNM stage of CRC OS in primary cohort. **A** All patients; **B** TNM stage I, II and III; **C** TNM stage I and II; **D** TNM stage III and IV; **E** TNM stage I; **F** TNM stage II; **G** TNM stage III; **H** TNM stage IV. Notes: CRC, colorectal cancer; OS, overall survival; TNM stage, tumor-node-metastasis stage

After successfully constructing the nomogram model, we incorporated it into the nutrition-related prognostic prediction of patients with CRC. We performed Kaplan–Meier curves for each TNM stage and the nomogram score could well stratify the survival outcome of those patients with stages I-IV, stage I-III, stage I-II, stage III-IV, stage I (all with low nomogram score), stage II, stage III, and stage IV (all with high nomogram score) (Fig. 6). Meanwhile, we also performed the Kaplan–Meier curves for each TNM stage in the internal validation cohort and found that the nomogram score showed consistent results with the primary cohort (Additional file 4).

## Discussion

Accurate prediction of prognosis plays an essential role in the management of CRC, as it may help determine the type, timing, and population of treatment [12]. Considering the high incidence of malnutrition in patients with CRC, we hypothesized that the nutritional status of CRC might affect the survival status of patients. In previous studies, TNM stage cannot distinguish patients with CRC TNM stages I, II, and III, and it had a poor survival prediction of patients with early-stage CRC [20]. Nutritional status has also been reported as a crucial factor in predicting cancer risk [21]. Thus, we aimed to develop a prospective nutrition-related model for predicting the short-term prognostic outcomes of CRC by combining clinical and nutritional indicators.

In this study, we constructed and validated a prognostic model based on TNM stage and nutritional parameters, which included TNM stage, radical resection, reduced food intake, activities and function declined, and albumin. This model can predict the short-term and long-term survival of CRC patients well. Currently, the TNM staging system for CRC is a widely used and practical predictor of OS and treatment choice [22]. However, TNM stage does not distinguish patients' short-term survival, so our model can make up for this deficiency. We all know that cancer patients, especially gastrointestinal tumors, often face malnutrition problems. Malnutrition in patients can affect the short-term survival and quality of life of patients. Among the nutritional parameters we picked included reduced food intake, activities and function declined, and albumin. Reduced food intake and activities and function declined are essential indicators in evaluating malnutrition, and they could reflect a body’s condition. Reduced food intake is a common symptom in patients with cancer, caused by a series of metabolic changes or tumors [23]. The reduced food intake of patients with cancer is caused by primary anorexia, and symptoms can be exacerbated by secondary nutritional effects. The simultaneous occurrence of high metabolism, high catabolism, and low anabolism aggravates the related weight loss and is caused by systemic inflammation and catabolism factors, which can partly act through the central nervous system. The combination of these dietary and metabolic factors leads to clinically recognized cachexia syndrome [24]. Loss of skeletal muscle mass is the main manifestation of cancer cachexia and is associated with reduced quality of life, progressive impairment of function, and worsening prognosis. Muscle atrophy causes weakness, activities and function declined, and fatigue in patients with cachexia and may increase the risk of respiratory failure, which is a common cause of death from cancer [25]. It seems that the two prognostic factors can better reflect the short-term condition of the body, especially when the patient’s nutritional status is poor. Poor nutritional status is often manifested as multifactorial physiological damage, such as aging and high comorbidities, leading to immune dysfunction, muscle atrophy, and poor quality of life. Malnutrition is associated with a decline in general functional status, delayed post-surgery recovery, high hospitalization, and increased mortality [26]. Serum albumin represents the nutritional and inflammatory status of the human body [27, 28]. Cancer-related malnutrition is a multimodal process, because many factors collude with each other; impair food intake; increase energy and protein requirements; reduce synthetic stimuli, such as physical activity; and change the metabolism of different organs or tissues. The multimodal drivers of malnutrition constitute the reason for using multiple treatment strategies to prevent, delay, or treat malnutrition in patients with cancer [29]. Previous studies have shown that serum albumin is an independent prognostic factor in CRC, and hypoalbuminemia is associated with poor survival in CRC [7, 30, 31]. The decrease in serum albumin level can directly reflect the increase in protein consumption and the demand of patients with cancer. A recent report by Evans et al. suggested that serum albumin should be considered a chronic disease characterized by inflammation, which is closely related to the risk of poor prognosis for patients, and the serum albumin concentration is reduced when inflammation is present [28].

Nomograms are considered a more reliable tool to quantify cancer risk than the traditional TNM staging system classification [32]. Previous studies have developed nomograms to predict the clinical outcomes of patients with CRC [33–35]. Similarly, in our study, we also found that the nomogram model we built had the highest C-index (0.74) and good calibration plot consistency. Meanwhile, the C-index, time-dependent ROC curves, and DCA indicated that the nomogram showed higher discrimination, prognostic prediction ability, and clinical benefit than the other models. We hypothesized that our model integrating TNM staging and nutritional parameters could improve the survival prediction performance of CRC patients. In addition, the scoring model we constructed provides accurate prediction performance for patients with cancer. Interestingly, in the survival analysis of different tumor stages, we found that the nomogram scores of patients with stage I were all at low risk and those with stage IV were at high risk, showing good predictive power in other subgroups of different combinations. We hypothesized that the nomogram score could accurately stratify the survival outcome of low-risk and high-risk patients.

Although the nomogram model could accurately predict the OS of patients with CRC, there still exist some limitations in the present study. First, we only included PGSGA as a nutritional prognostic tool, and there may be a better tool for assessing the nutritional status of CRC. Second, declined food intake and activities and function declined were only generated through patient self-survey questionnaires, lacking more systematic and professional assessment methods. Thirdly, our analysis is only a cross-sectional study, and dynamic monitoring of patients is required. In addition, some factors that could also contribute to short-term survival of patients need to be further considered, such as patients' cachexia status and inflammation level. Finally, we developed and validated a prognostic nomogram for CRC using this multicenter database. Future studies may require external validation cohorts to verify this finding.

## Conclusions

In our study, nutrition-related and commonly used clinical parameters were integrated to construct a CRC prognostic nomogram. The present nomogram model had a better prognostic prediction ability than the traditional TNM stage, PGSGA, and TNM + PGSGA models. The scored nomogram could be a better tool for predicting the prognosis of patients with CRC at each TNM stage. Notably, the nomogram might be a novel and practical tool for evaluating the short-term outcomes of OS in patients with CRC.

## Supplementary Information

**Additional file 1:** Flowchart of patient selection for this study.

**Additional file 2:** The cut-off value of nomogram score.

**Additional file 3: Table S1.** Table Comparison of C-index among different prognostic models

**Additional file 4:** Risk group stratification within each TNM stage of CRC OS in internal validation cohort. (A) All patients; (B) TNM stage I, II and III; (C) TNM stage I and II; (D) TNM stage III and IV; (E) TNM stage I; (F) TNM stage II; (G) TNM stage III; (H) TNM stage IV. Notes: CRC: Colorectal Cancer; OS: Overall Survival; TNM stage: Tumor-Node-Metastasis Stage.

## Data Availability

The datasets used and/or analysed during the current study are available from the corresponding author on reasonable request.
