# Supplementary material for: A novel nutrition-related nomogram for the survival prediction of colorectal cancer-results from a multicenter study
Source: Nutr Metab (Lond). 2023 Jan 4;20:2. doi: 10.1186/s12986-022-00719-8 (PMC9814216; doi:10.1186/s12986-022-00719-8)
Supplement: Supplementary file 1 — Additional file 1: Flowchart of patient selection for this study. [file 12986_2022_719_MOESM1_ESM.pdf]

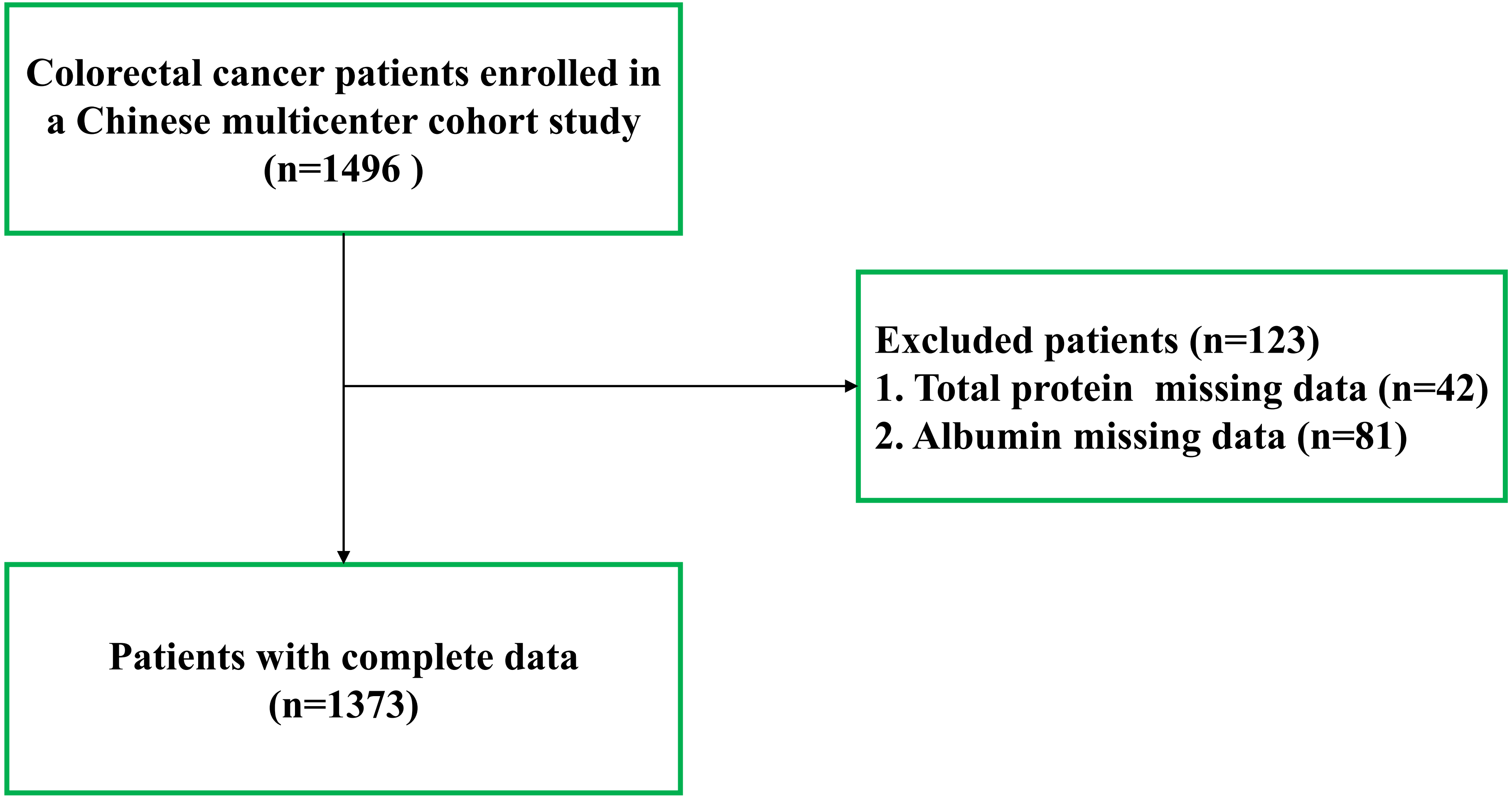
Colorectal cancer patients enrolled in a Chinese multicenter cohort study (n=1496)
Excluded patients (n=123)
1. Total protein missing data (n=42)
2. Albumin missing data (n=81)
Patients with complete data (n=1373)
