# Supplementary figures and images for: A novel nutrition-related nomogram for the survival prediction of colorectal cancer-results from a multicenter study
Source: Nutr Metab (Lond). 2023 Jan 4;20:2. doi: 10.1186/s12986-022-00719-8 (PMC9814216; doi:10.1186/s12986-022-00719-8)

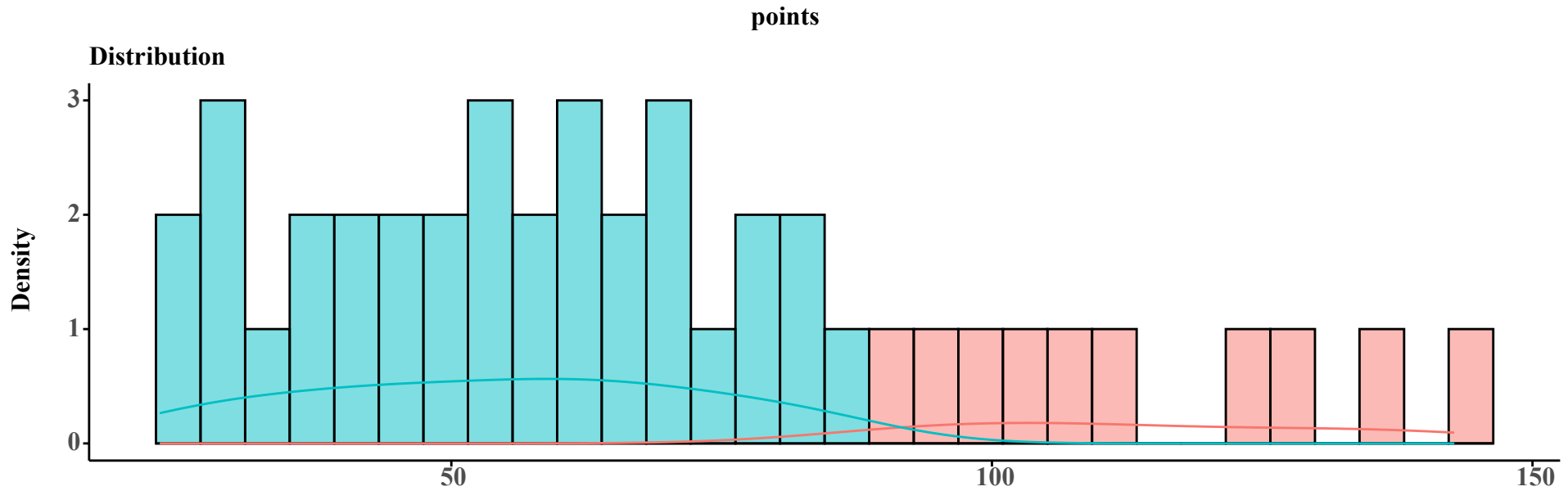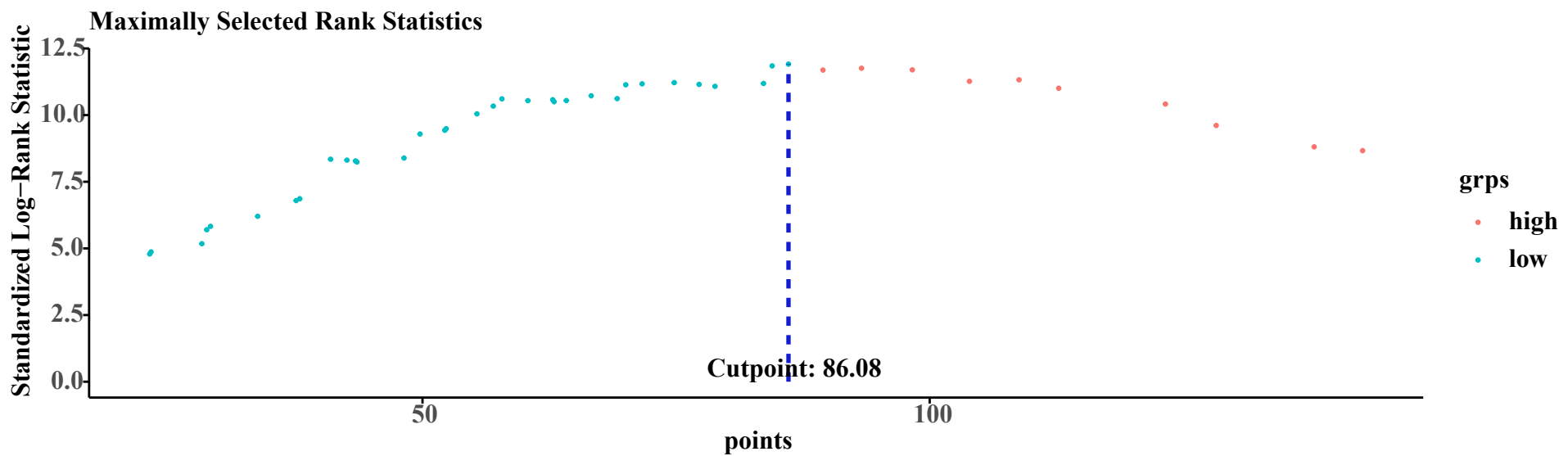

Supplement: Supplementary file 2 — Additional file 2: The cut-off value of nomogram score. [file 12986_2022_719_MOESM2_ESM.pdf]
