# Supplementary material for: A novel nutrition-related nomogram for the survival prediction of colorectal cancer-results from a multicenter study
Source: Nutr Metab (Lond). 2023 Jan 4;20:2. doi: 10.1186/s12986-022-00719-8 (PMC9814216; doi:10.1186/s12986-022-00719-8)
Supplement: Supplementary file 3 — Additional file 3: Table S1. Table Comparison of C-index among different prognostic models [file 12986_2022_719_MOESM3_ESM.docx]

**Table S1 Table Comparison of C-index among different prognostic models**

| Models | Primary cohort | Comparable  *P* value | Internal validation cohort | Comparable  *P* value |
| --- | --- | --- | --- | --- |
|  | C-index (95%CI) |  | C-index (95%CI) |  |
| Model 1 (Nomogram) | 0.74(0.72-0.77) | - | 0.75(0.70-0.80) | - |
| Model 2 (TNM stage) | 0.70(0.67-0.72) | <0.001 | 0.68(0.63-0.73) | <0.001 |
| Model 3 (PGSGA) | 0.58(0.55-0.61) | <0.001 | 0.59(0.53-0.64) | <0.001 |
| Model 4 (TNM stage and PGSGA) | 0.73(0.67-0.76) | 0.004 | 0.71(0.66-0.77) | 0.026 |

Notes: C-index: Concordance index; 95%CI: 95% Confidence interval; TNM stage: Tumor-node-metastasis stage; PGSGA: Patient Generated Subjective Global Assessment; C-index: concordance index.

Model 1: Nomogram (TNM stage, radical resection, reduced intake, activities and function declined, and albumin);

Model 2: TNM stage;

Model 3: PGSGA;

Model 4: TNM stage and PGSGA;
