# Supplementary material for: A novel nutrition-related nomogram for the survival prediction of colorectal cancer-results from a multicenter study
Source: Nutr Metab (Lond). 2023 Jan 4;20:2. doi: 10.1186/s12986-022-00719-8 (PMC9814216; doi:10.1186/s12986-022-00719-8)
Supplement: Supplementary file 4 — Additional file 4: Risk group stratification within each TNM stage of CRC OS in internal validation cohort. (A) All patients; (B) TNM stage I, II and III; (C) TNM stage I and II; (D) TNM stage III and IV; (E) TNM stage I; (F) TNM stage II; (G) TNM stage III; (H) TNM stage IV. Notes: CRC: Colorectal Cancer; OS: Overall Survival; TNM stage: Tumor-Node-Metastasis Stage. [file 12986_2022_719_MOESM4_ESM.pdf]

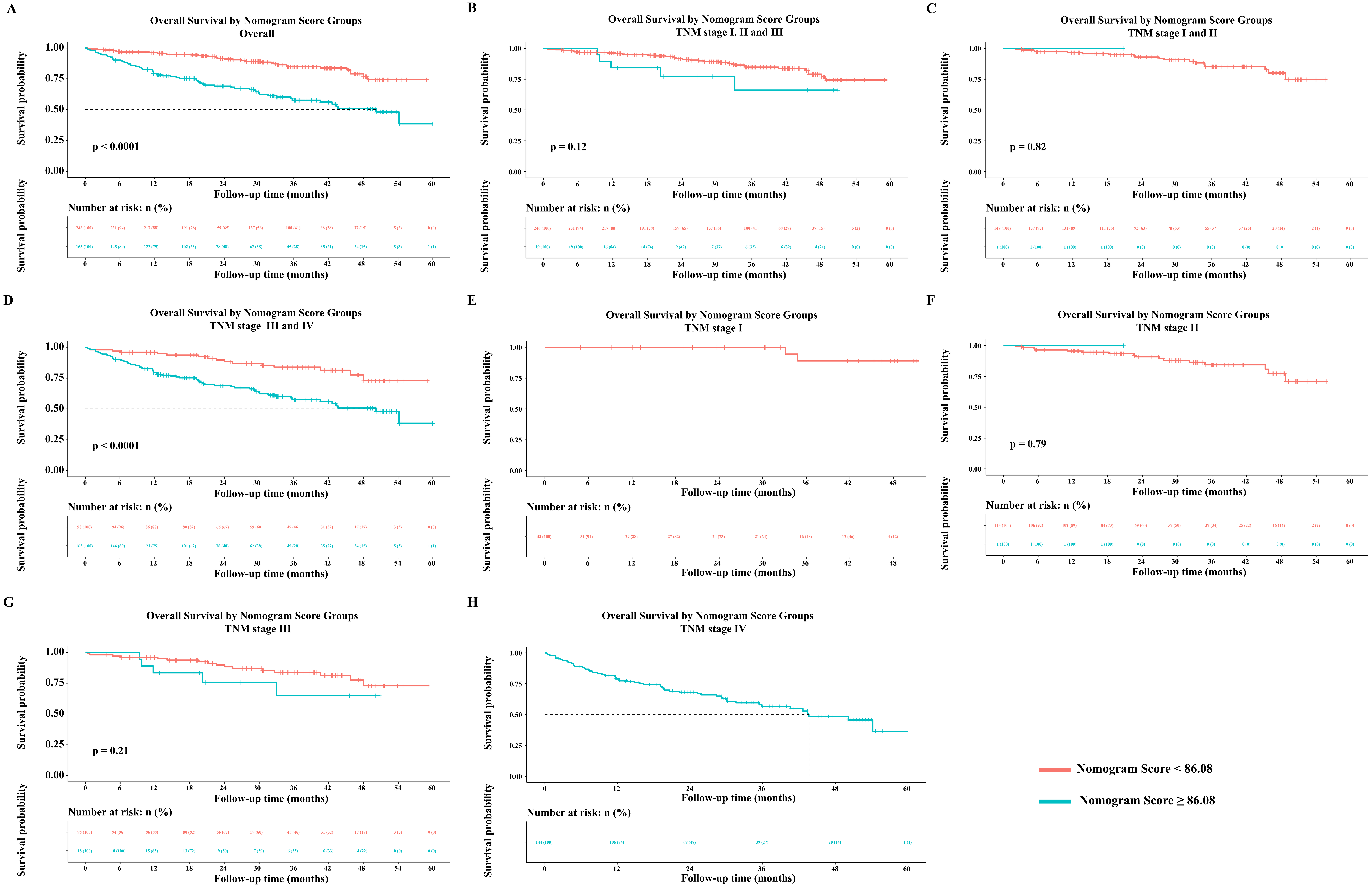
A
Overall Survival by Nomogram Score Groups
Overall
p < 0.0001
B
Overall Survival by Nomogram Score Groups
TNM stage I. II and III
p = 0.12
C
Overall Survival by Nomogram Score Groups
TNM stage I and II
p = 0.82
D
Overall Survival by Nomogram Score Groups
TNM stage III and IV
p < 0.0001
E
Overall Survival by Nomogram Score Groups
TNM stage I
F
Overall Survival by Nomogram Score Groups
TNM stage II
p = 0.79
G
Overall Survival by Nomogram Score Groups
TNM stage III
p = 0.21
H
Overall Survival by Nomogram Score Groups
TNM stage IV
Survival probability
Follow-up time (months)
Number at risk: n (%)
Nomogram Score < 86.08
Nomogram Score ≥ 86.08
